# Tuberculosis—Its Origin and Dissemination

**Published:** 1902-03

**Authors:** James N. Brawner

**Affiliations:** Atlanta, Ga.


### TUBERCULOSIS—ITS ORIGIN AND DISSEMINATION.*

BY JAMES N. BRAWNER, M.D.,

ATLANTA, GA.

*Mr. President, Ladies and Gentlemen:*

It has been estimated that one death in seven is caused from tuberculosis. In the city of Atlanta there occurs, approximately, 425 deaths annually from consumption. In Philadelphia the annual death rate from tuberculosis is 2,400, and in the United States probably about 200,000.

From these figures it will be seen that tuberculosis, with the long course which the disease usually runs, causes more suffering than all the other infectious diseases combined. No wonder the world is rising in arms to fight the tubercle bacillus—the little germ that thus destroys so many lives.

This united fight should be waged more and more till every one of these little pests have been destroyed, if such a thing be possible. Nothing will aid so much to this end as for the general public to be educated in the origin and dissemination of the disease.

At this day and time most people know that tuberculosis is caused by a germ—the tubercle bacillus—a little vegetable microorganism about one three-thousandth of an inch in length. You see before you on the chart the appearance of the germ as it is seen under the microscope when properly stained. You see it is a little rod-shaped organism, sometimes slightly curved. In good soil this organism multiplies very rapidly by a process known as fission. The germ grows until it gets a certain size, and then a constriction can be seen to begin in the center, which becomes more and more marked until cleavage is complete, as is represented to you in the diagram, in different stages.

This diagram shows the constriction beginning to take place; this one shows it more marked; this one its completion, forming

* Read before the Georgia State Sociological Society at the first annual meeting held in Atlanta, December 13, 1901.

two germs. Each of these germs will now undergo division, thus increasing in a geometrical ratio. Under favorable conditions these organisms multiply so rapidly that one germ can produce thousands in twenty-four hours.

Now take a person with tuberculosis of the lungs, in other words, consumption, with cough and expectoration. The sputum of such an individual fairly swarms with these germs. In fact, it has been estimated that a person with well marked consumption expectorates, on an average, about 70,000,000 of these organisms daily. Now, suppose this individual expectorates about promiscuously on the floor, walls of the room, sidewalks and other public places. The sputum dries and becomes powdered into dust, which floats about in the air.

In the room of a consumptive, if he is allowed to expectorate on floor or ceiling, it is plain that the dried sputum soon becomes dust, which floats around in the room to be inhaled by other individuals. Now, the dust inhaled, which contains large numbers of living tubercle bacilli, may lodge in the throat, get into the lymph channels or lymph glands, and thence into the general circulation, to lodge in various portions of the body; or they may be inhaled into the smaller bronchi or air cells, get into their walls and begin to multiply. It is probably possible that dust can be inhaled as far down as the smallest of these bronchioles. If it is, then it is possible to inhale these little germs just as far.

After they are inhaled into the lungs and they get into the walls of the bronchioles or air-cells, one of two processes takes place, depending altogether upon the resistance of the lung tissue in which they are lodged: if the resistance of the lung tissue is so great that they cannot grow and multiply, the germs die and do no damage; but if the resistance is poor the germs can grow rapidly, and quickly cause breaking down of the lung tissue.

In the diagram is shown the lung tissue breaking down in two different stages. In the right lung the process is shown as just beginning. The lung tissue at this place is solid. At this stage a person may have consumption and never suspect it, as there may be no cough, and the person may feel perfectly well. Furthermore, at this stage very few germs, if any, are thrown off with the sputum, as softening and breaking down of the lung tissue has not begun. But when the process extends, the lung tissue begins to soften down, and soon a condition is found which is represented to you in the left lung, diagramatically, of course.

When a person has consumption at this stage is when he is the greatest source of danger to the community. He walks about, thinking perhaps, that he only has a protracted cold, expectorating here and there on the floor, ceiling, side-walks and other public places, literally scattering millions of living germs all in the community wherever he visits.

At this stage of consumption a person is a veritable source of danger to those about him. When a little sputum from such an individual is properly stained and examined under the microscope, it looks something like what you see in this diagram. The germs are shown as red, rod-shaped organisms. Whenever these germs are found in a person's sputum, he has consumption without any doubt. This is an absolutely sure sign.

Now, when it is remembered that several million people are afflicted with consumption in the United States, each expectorating several millions of these little pests daily, it can be plainly seen that every one of us breathe more or less of these germs, at some period during our lives.

Now the question may be asked, Why is it, if it be true that all of us are exposed to these germs, inhale them where they get into the very tissue of our lungs, that all of us do not die from consumption?

The fact is this: That all of us do get these organisms into our lungs, but those of us that have been so fortunate as to inherit good, sound and resistant lung tissue, and have been prudent enough to keep it in a healthy condition, are able to resist the action of the germs. In other words, they cannot grow in such healthy lung tissue. However, if tubercle bacilli get into a lung where the tissue is weak in resistance, they begin to grow and multiply and soon cause the destruction of more or less lung tissue. Weak, non-resisting lungs may either be inherited or acquired. In the former, through the well known laws of heredity, the child becomes to possess a poor quality of lung tissue, and this fact is often demonstrated by several members of the same family dying of consumption.

Whether a child is ever born with consumption—that is, with the tubercle bacilli in its lungs—is a disputed fact. Even if it does occur, however, it is very rare.

By bad habits, by living under unhygienic conditions, by excesses of all sorts, a person who has inherited the best of lungs may contract and die with consumption, where they are exposed to it very much. This is often seen in the case of husbands and wives. Suppose a husband dies of tuberculosis, the wife, through worry, loss of sleep, and overwork, becomes run down, and gets in low health. Very frequently in two or three years she, too, dies of the same trouble, although, to begin with, she may have had good, resistant lungs; but the low health lessens their resistance, and the germs she has breathed in the dust from the dried sputum of her husband, lodge in good soil to grow unmolested.

Tuberculosis is also probably sometimes contracted by close communication with tuberculous patients where small, moist doplets of sputum are expelled in coughing.

Another source of infection is through milk and meat that contain living tubercle bacilli. For this reason, cows and hogs should be rigidly inspected for this disease. The communicability of bovine tuberculosis to man has recently been called in question, but

To prevent the spread of tuberculosis is a question that is occupying the minds of the greatest men of to-day. That it is a problem hard to solve is a question beyond doubt.

Much good, however, is being accomplished where systematic regimen is being carried out. Many requisites are necessary to do much in lessening or to stamp out tuberculosis. Some are as follows:—

1. We should try to raise a people that is strong and healthy, by prohibiting, as far as possible, the marriage of people with consumption, or a predisposition thereto.

2. People should be educated how to live—an object for which this Society has been formed; they should be taught not only how to make money, but how to preserve their health, both mental and physical.

3. People should be taught the nature of consumption, so that they may have rational ideas of its causation. Suppose every person in the United States knew that the sputum from a consumptive contains millions of these little deadly pests; that this sputum dries into dust and is inhaled by themselves and by others, and that this is the way it is more frequently communicated? Wouldn't people be more careful as to where and how they expectorated? The consumptive himself would burn or disinfect his sputum to protect his family and friends.

4. Expectoration should be prohibited in all public places. In such places cuspidors containing an antiseptic should be conveniently placed. Decency alone should prevent expectorating on the floor or walls. But the lower classes or not so decent, consequently they should first be educated as to the danger of such promiscuous spitting.

5. The sputum of all consumptives, wherever possible, should be burned. One of the best means for this is the use of little paper cuspidors, which are cheap, and can be burned after use. If burning is not feasible, it should be disinfected by various chemicals.

But in dealing with consumption it is easier to tell what to do than to do it. When we remember that there are probably 5,000,000 people in the United States affected with this disease, half of them not aware of the fact, we can begin to see the magnitude of the problem.

By what means can we prevent the consumptive not aware of his condition from expectorating promiscuously about, especially when he is not aware of the danger it entails?

From the very nature of the disease it can be plainly seen that any process by which we may attempt to lessen its frequency must be a slow one. City, county and State authorities should prevent spitting in public places. All kinds of public buildings and institutions should prevent it. In hospitals every particle of sputum should be burned, and special hospitals should be built for consumptives. Colonization of tuberculous patients should be done wherever feasible.

But the main hope of our people to-day is the inheritance of a sound constitution and its maintenance by living under hygienic conditions. A consumptive should never be allowed to marry if it can possibly be prevented.

But reforms, to be permanent, must be slow. The germ of consumption and the condition which favors its growth—that is, sorry lungs—must be exterminated *pari passu;* they must go together. The people with weak lungs must gradually be replaced by a people with strong lungs; then tubercle bacilli will get fewer and fewer, and possibly, at some remote date, consumption may be a disease of the past.

